# Neuromimetic model of saccades for localizing deficits in an atypical eye-movement pathology

**DOI:** 10.1186/1479-5876-11-125

**Published:** 2013-05-22

**Authors:** Pierre M Daye, Lance M Optican, Emmanuel Roze, Bertrand Gaymard, Pierre Pouget

**Affiliations:** 1 Laboratory of Sensorimotor Research, National Institutes of Health, Bethesda, Maryland; 2 Pierre et Marie Curie Paris-6 University, INSERM UMRS 975, CNRS 7225, Paris, France; 3 Assistance Publique Hôpitaux de Paris (AP-HP), Department of Neurology, Pitié-Salpêtrière Hospital, Paris, France

## Abstract

**Background:**

When patients with ocular motor deficits come to the clinic, in numerous situations it is hard to relate their behavior to one or several deficient neural structures. We sought to demonstrate that neuromimetic models of the ocular motor brainstem could be used to test assumptions of the neural deficits linked to a patient’s behavior.

**Methods:**

Eye movements of a patient with unexplained neurological pathology were recorded. We analyzed the patient’s behavior in terms of a neuromimetic saccadic model of the ocular motor brainstem to formulate a pathophysiological hypothesis.

**Results:**

Our patient exhibited unusual ocular motor disorders including increased saccadic peak velocities (up to ≈1000 deg/s), dynamic saccadic overshoot, left-right asymmetrical post-saccadic drift and saccadic oscillations. We show that our model accurately reproduced the observed disorders allowing us to hypothesize that those disorders originated from a deficit in the cerebellum.

**Conclusion:**

Our study suggests that neuromimetic models could be a good complement to traditional clinical tools. Our behavioral analyses combined with the model simulations localized four different features of abnormal eye movements to cerebellar dysfunction. Importantly, this assumption is consistent with clinical symptoms.

## Introduction

Ocular flutter is an abnormal eye movement consisting of repetitive, irregular, involuntary bursts of horizontal saccades without an intersaccadic interval [1]. It is generally superimposed on normal ocular motor behavior and its occurrence may be facilitated by various events, such as blinks, the triggering of normal saccades or optokinetic stimulation [2,3] and has been observed during pursuit [4]. The physiology of this rare disorder remains unclear. It probably results from a dysfunction of brainstem ocular motor structures, in particular the paramedian pontine reticular formation (PPRF) involved in saccade generation: excitatory burst neurons (EBN), inhibitory burst neurons (IBN) and omnipause neurons (OPN). EBN drive the ipsilateral motor neurons, IBN inhibit the contralateral motor neurons, and OPN keep EBN and IBN silent, except immediately before and during saccade execution. Earlier hypotheses ascribed saccadic oscillations to impaired OPN function [5]. More recently, instability in positive feedback loops involving EBN and IBN has been hypothesized as the critical factor responsible for saccadic oscillations [6]. Oscillations could be generated in these positive feedback loops if neurons have a post-inhibitory rebound (PIR), a spontaneous burst of activity following the end of a sustained inhibition [4,6]-[8]. The oscillations observed during pursuit [4] could also be linked to the OPN discharge, because it is known that OPN activity is decreased during pursuit [9].

Here, we report the case of a patient with a flutter and very atypical saccade impairments. Saccades in our patient showed four abnormal characteristics. First, leftward saccades had an increased peak velocity while rightward saccades had a peak velocity close to normal. Second, saccades contained a dynamic overshoot: saccade trajectories reversed at the end of the movement. Third, at the end of a saccade, there was an asymmetrical centripetal drift, more pronounced after rightward saccades. Finally, our patient generated saccade-induced oscillations. The neural basis of most of these ocular motor symptoms is correctly understood individually. We sought to determine if their coexistence within a single patient implies widespread lesions or dysfunctions, or whether they could result from the alteration of a focal neural structure. We focused on a neuromimetic saccadic model of ocular motor control that integrates the current knowledge of the ocular motor brainstem, superior colliculus and cerebellar circuitry. The fit of the model to the kinematics of the saccades was determined by four parameters that are each a function of the saccadic displacement: the maximum collicular discharge, the maximum contralateral cerebellar discharge, the maximum ipsilateral cerebellar discharge and the timing of the onset of that discharge.

## Methods

### Patient clinical history

An 18-year-old female with non-consanguineous parents had no familial history of neurologic or psychiatric disorder. Her pregnancy, childbirth and perinatal period were normal. She had a mild learning disability from age eight. At age 14 she started to complain of visual disturbances that were attributed to abnormal eye movements. Her neurological condition gradually deteriorated over the next four years: she developed dysarthria, then behavioural changes and cognitive deterioration, and eventually mild gait disturbances.

On examination at age 18, she had a mild intellectual disability associated with social withdrawal and depressive features. Motor and speech examination suggested a diffuse central nervous system dysfunction with the combination of a cerebellar syndrome, an akineto-rigid parkinsonism associated with multifocal dystonia and a pyramidal syndrome without motor deficit. Eye movement recordings revealed atypical saccadic eye movements with asymmetrical peak velocity and post-saccadic drift, a dynamic overshoot and an ocular flutter. These ocular motor deficits are quantified in the results.

Repeated work-ups failed to identify an immunologic disorder; particularly we failed to detect any paraneoplastic antibodies and a comprehensive search for a tumor remained negative. She had no improvement following a therapeutic trial with intravenous Immunoglobulin therapy. Clinical evolution, MRI of the brain and spinal cord, and analysis of cerebrospinal fluid were normal, ruling out multiple sclerosis. Dopamine transporter imaging with 123I-FP-CIT (DaTSCAN) showed a bilateral reduction in striatal uptake consistent with a dysfunction of the nigrostriatal pathways. The neurometabolic investigations found no abnormality and genetic investigations were negative for Huntington disease, DRPLA, fragile X, Friedreich ataxia, dominant spinocerebellar ataxias, PLA2G6, GFAP. Neurologists concluded that she had a probable heredo-degenerative disorder of unknown origin.

### Paradigm and data acquisition

The patient gave an informed consent before the study. All the procedures were approved by the local ethics committee (CERES: Comité d’évaluation éthique des projets de recherche en santé. N 2012-25) and conducted in conformity with the Declaration of Helsinki. She was tested on three different dates. The first two sessions were separated by six months, the second and the third sessions were separated by three months. The subject sat 57 cm from a screen in a completely dark room. A gap protocol was used during these sessions. Briefly, a central fixation point (0.5 deg diameter, green) appeared for 2800, 3500 or 4000 ms then disappeared for 200 ms (gap period). After the gap, a target was presented either leftward or rightward (controlled pseudo-random sequence). 12 targets were presented during a recording block. A single block was presented during the first session, four blocks during the second session and two during the last session. Of the seven blocks, one used variable amplitudes between 5 and 20 deg, the others used a fixed amplitude of 25 deg. During the second session, one block of 16 trials with upward and downward target presentation (no gap) was presented to the patient. The same number of upward and downward target positions were presented in a randomized order. Therefore, we had six blocks with horizontal target displacements and one block with vertical target displacements.

The target was presented using a personal computer running meyePARADIGM (e(ye)BRAIN SA, Paris, France). Horizontal and vertical monocular eye positions were acquired using an IVIEW X HI-SPEED (SensoMotoric Instruments, GmbH, Germany) video eye-tracker at 500 Hz. The data were low-pass filtered at 50 Hz. Saccades were detected using a generalized likelihood ratio (GLR) algorithm as in [10,11]. Every trial was visually inspected; a manual correction of the detection parameters was applied if a saccade was not detected. 220 leftward (39 of them come from the variable amplitude paradigm) and 251 rightward (21 of them come from the variable amplitude paradigm) valid saccades were analyzed.

### Comparison of linear regression slopes

We used the test detailed in [12] to compare the slopes of two linear regressions:

(1) $$\begin{array}{ll} Y_{a} & \;=\;\;\alpha_{a}X+\beta_{a}\; \end{array}$$

(2) $$\begin{array}{ll} Y_{b} & \;=\;\;\alpha_{b}X+\beta_{b}\; \end{array}$$

(3) $$\;\begin{array}{ll} t & \;=\;\;\frac{\alpha_{a}-\alpha_{b}}{\sqrt{\text{SEM}{\left(\alpha_{a}\right)}^{2}+\text{SEM}{\left(\alpha_{b}\right)}^{2}}}\; \end{array}$$

*α*_*a*_ and *α*_*b*_ are the slopes of the regressions (1) and (2). *β*_*a*_ and *β*_*b*_ are the intercepts of the regressions. *X* represents the independent variable while *Y*_*a*_ and *Y*_*b*_ represent the dependent variables. Equation (3) computes the t-statistic value used to test the difference between *α*_*a*_ and *α*_*b*_. SEM in eq. (3) corresponds to the standard error of the mean.

### Model

In the following subsections, we will describe the different parts of the model and how they affect the saccadic eye-movement behavior.

#### General structure

**Figure 1:**
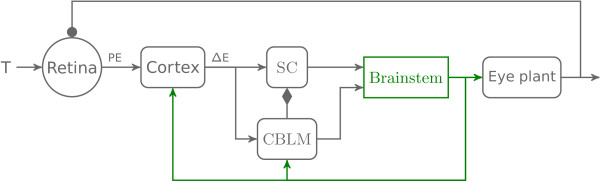
**General architecture of the model.** This figure represents the different parts of the model and their feedforward and feedback connections. First, a target (T) appears on the retina and generates a position error (PE) sent to the cortex. Second, the cortex computes the desired eye displacement (*Δ*E) and sends it to the superior colliculus (SC) and the cerebellum (CBLM). Third, SC and CBLM send a drive to the brainstem which sends back an efference copy of the eye position to CBLM which controls eye displacement. CBLM also modulates the collicular activity through a facilitation signal. Finally, the brainstem sends a drive to the eye plant. Lines with arrowheads correspond to excitation, with filled circles correspond to inhibition, and with filled diamonds correspond to facilitation. Details of the brainstem connectivity are presented in Figure 2.

Figure 1 shows the unilateral general organization of the model, including the feedback loops. The overall architecture is similar to [13,14]. Briefly, based on target visual information, the cortex determines the desired eye displacement and sends it to the superior colliculus (SC) and to the cerebellum (CBLM). The superior colliculus sends a drive to the brainstem that shapes the initial eye velocity while the cerebellum controls eye trajectory through a drive sent to the brainstem. The CBLM modulates the amplitude of the collicular discharge through a disfacilitation signal (diamond tipped arrow in Figure 1). The brainstem sends the motor signal to the eye plant as well as an efference copy of this signal back to the cerebellum and to the cortex (green arrows from the brainstem to CBLM and Cortex in Figure 1). At the end of the saccade, the cortex evaluates whether a visual position error remains and triggers a corrective saccade if needed.

**Figure 2:**
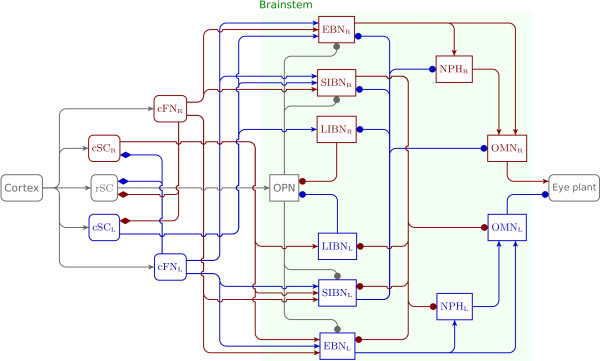
**Feedforward bilateral connectivity.** This figure represents a detailed view of the bilateral architecture with feedforward connections. Gray boxes correspond to neural structures modeled as being unilateral. Red boxes represent right side neural structures. Blue boxes represent left side neural structures. The green shadow box represents the detailed description of the green box “brainstem” in Figure 1. Lines with arrowheads correspond to excitation, with filled circles correspond to inhibition, and with filled diamonds correspond to facilitation. No efferent-copy signals (to cortical and/or subcortical neural areas) are represented in this figure because they correspond to feedback connections.

#### Feedforward bilateral architecture

The model presented in this paper focuses on a detailed representation of the brainstem. Figure 2 shows the bilateral feedforward connectivity included in the model from the cortex to the eye plant. Gray items represent neural structures modeled as being unilateral (without any loss of generality). Red items represent right side neural structures while blue items represent left side neural structures. Dot-tipped lines correspond to inhibitory connections, arrow-tipped lines represent excitatory connections, and diamond-tipped lines represent disfacilitation signals. Importantly, we divide the IBN into two populations, one with a long lead and one with a short lead, and connect them differently [15]. Note that the model is bilateral but we only considered the movement of the right eye. This section describes the different neural areas outside the brainstem.

In the model, the simplified cortex computes the amplitude of the saccadic displacement needed to make a saccade towards a visual target. The cortex block has four inputs: an estimate of the orientation of the eye, an internal estimate of eye velocity, the retinal position of the target (delayed by 150 ms to account for primary visual cortex computations, the extraction of the target position and the programming of the desired saccade amplitude) and an input from cerebellum to signal when the saccade is over. The model of the cortex includes a refractory period of 50 ms (starting when the eye velocity drops under 20 deg/s) during which no new saccade can be triggered. New saccades are triggered if the visual error is larger than one degree. Finally, the input-output gain between the spatial position of the target on the retina and the amplitude of the saccade is equal to 0.9 to reproduce saccadic undershoot behavior of healthy subjects [16]. Then, the model of the cortex computes the desired amplitude of the saccade and sends this information downstream to the superior colliculus and the cerebellum.

The superior colliculus (SC) is divided in the model into three subparts: a rostral part corresponding to very small errors and two caudal parts (left and right) that model the combined activity of the collicular burst and buildup neurons. There is no collicular discharge in the caudal part of the modeled SC when the desired gaze displacement is smaller than a threshold value of one degree (output of cSC_L_ and cSC_R_ is equal to zero and rSC is discharging at its peak in Figure 2). The rSC corresponds to the rostral pole of the SC initially observed by Munoz and Wurtz [17] but must be seen as a simplification of the actual SC circuitry [18]. In more caudal recordings, Wurtz and Goldberg [19] have shown that the activity of the SC deep layers is related to a particular displacement (orientation and amplitude) of the eye. The rSC in the model receives three inputs: a disfacilitation signal from each caudal fastigial nucleus and the desired eye displacement from the cortex. Both caudal superior colliculi receive two inputs: the desired eye displacement and a disfacilitation signal from the contralateral caudal fastigial nucleus. The output of the caudal collicular parts is saturated to account for the saccadic peak velocity saturation with increasing saccadic amplitudes. The maximum discharge of the caudal superior colliculus (*cSC*_*m*_) is determined by a piecewise linear function that uses the desired saccadic displacement as input. This function was manually tuned before the simulations. The amplitude of the collicular discharge shapes the initial acceleration of saccadic eye movement.

The cerebellum is the core of the saccadic controller in the proposed model. It has three different roles; it controls the trajectory to ensure that the saccade ends close to the target, it modulates the level of activity of the SC and it stops the saccade by sending a choke signal to the contralateral long-lead inhibitory burst neurons (LLIBN). The output of the cerebellum is represented in the model by the discharge of the caudal fastigial nuclei. Each nucleus has two inputs: the desired eye displacement and an efference copy representing eye velocity [20]-[22]. Each nucleus has two different roles depending on the movement direction. The caudal fastigial nucleus contralateral to the saccadic displacement controls the movement, while the ipsilateral caudal fastigial nucleus stops the movement at the end of the saccade^a^. To control the trajectory, the contralateral caudal fastigial nucleus compares the desired eye displacement to an estimate of the current displacement obtained by integrating (in a mathematical sense) the efference copy of eye velocity and computes the appropriate drive to correct the trajectory. The amplitude of cerebellar discharge is saturated to reproduce the saturation of the peak velocity of saccades as a function of the saccadic amplitude, called the main sequence [23]. The maximum discharge of the caudal fastigial nucleus (*cFN*_*Max*_) is determined by a piecewise linear function using the desired saccadic displacement as input. The ipsilateral caudal fastigial nucleus discharges only at the end of the saccade to stop the movement. The timing of the activity onset (*iFN*_*o*_) and the intensity of the discharge (*iFN*_*Max*_) of the ipsilateral caudal fastigial nucleus is determined in the model by piecewise linear functions using the desired eye displacement as input. These functions were manually tuned before the simulations to ensure correct saccadic accuracy and match the main sequence relationship [23,24]. Finally, the cerebellum also modulates the collicular activity through a disfacilitation signal. This signal is proportional to the amplitude of the eye motor error. Several studies have shown the existence of an excitatory projection from the deep cerebellar nuclei (dCN) to SC in the rat [25]-[27] and in the grey squirrel [28] that can facilitate or disfacilitate collicular activity [27].

To summarize, the kinematics of the saccades were determined by four parameters that are each a function of the saccadic displacement: the maximum collicular discharge, the maximum contralateral cerebellar discharge, the maximum ipsilateral cerebellar discharge and the timing of the onset of the discharge. As previously explained, these parameters were tuned manually once for the healthy saccade case and once to reproduce the patient’s behavior.

The input-output relationship between the innervation of the ocular muscles and the movement of the eye is modeled as a second-order transfer function with two time constants (150 ms and 5 ms). It receives two inputs: one from the right motoneuron nucleus and a second from the left motoneuron nucleus.

#### Brainstem and neuron model

**Figure 3:**
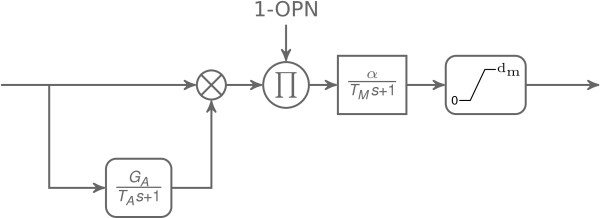
**Neuron model.** Three stages composed the neuron model used in this paper. The first one is a neuronal adaptation with a gain *G*_*A*_ and a time constant *T*_*A*_. In the second stage, this drive is modulated by the OPN activity. In the third stage, the modulated signal is sent to a first order transfer function (gain: *α*, time constant: *T*_*M*_) representing the membrane potential dynamics with a saturated output between 0 and *d*_*m*_≥0. The crossed circle represents a sum operator. The circled *π* represents a product.

The architecture of the brainstem connectivity used in the model is shown in Figure 2. The model architecture is an updated version of [6] which includes two new populations of neurons: the long-lead inhibitory burst neurons (LIBN_R_ and LIBN_L_) and the nuclei prepositus hypoglossi (NPH_R_ and NPH_L_). The activity of each brainstem neuronal population is represented in the model by the same neuron model, shown in Figure 3. This model combines a linear burster (as in [13,14]) and neuronal adaptation as in [6]. T_M_ represents the membrane time constant, *α* represents the gain of the neuron, G_A_ corresponds to the adaptation gain and T_A_ represents the adaptation time constant. The neuronal discharge is saturated between zero and *D*_*max*_. Finally, compared to [6], the OPN activity has a multiplicative inhibitory behavior on downstream neurons instead of an additive effect. Thus, when OPN are discharging, the activity at the input of the membrane low-pass filter is equal to zero.

The model divides IBN into two subpopulations (as reported in [15]): short-lead IBN (SIBN as in [6]) and long-lead IBN (LIBN). Scudder et al. recorded the two types of IBN in monkeys and divided them according to their lead time with respect to saccade onset (lead time of LIBN >15 ms) [15]. These authors reported that SIBN discharge was related to the saccade dynamics but could not be used to turn off the OPN because their activity was too late. As a conclusion, Scudder et al. proposed that LIBN could be used to turn off the OPN [15]. This assumption was recently confirmed [29] and used in our model. The modeled LIBN have two inputs: an excitation from the contralateral caudal superior colliculus and an inhibition from the contralateral short-lead inhibitory burst neurons (SIBN). LIBN inhibit the OPN during saccade execution. A horizontal eye movement is generated by the combined activity of SIBN driving contralateral NPH and OMN and EBN driving ipsilateral NPH and OMN.

The nucleus prepositus hypoglossi is a key structure in the brainstem to ensure that the eyes remain stationary between saccades [30,31]. It receives drives from the burst neurons, integrates them (in the mathematical sense) and projects to the ocular motor nuclei. Thus, NPH nuclei are part of the neural integrator for horizontal eye movements [32]. However, the ocular motor neural integrator is not perfect; in darkness it leaks with a time constant of approximately 20 seconds [33]. The model includes a leaky integrator with a time constant of 20 seconds to reproduce that behavior. In the model, NPH blocks have two inputs: an inhibition from the contralateral short-lead inhibitory burst neurons and an excitation from the ipsilateral excitatory burst neurons. Each nucleus projects to the ipsilateral oculomotor nucleus. Finally, it is known that the flocculus and the paraflocculus of the cerebellum are involved in the integration process; post-saccadic drifts with amplitude a function of the orbital position were reported following a lesion of the flocculus and paraflocculus [34]. It must be stressed that our model of the neural integrator does not include an orbital-dependent drift because this level of complexity is beyond the scope of this paper.

#### Simulations

All the simulations in this paper were performed on a personal computer running MATLAB/SIMULINK (The Mathworks, Natick, MA, USA).

## Results

### Patient behavior

To test the sensorimotor eye movement function of a patient, the first paradigm traditionally used is the visually guided saccade test. During this protocol, targets were presented in sequence at different places and the patient was asked to look at the target as soon as she saw it. Several variations of this protocol exist, depending on the timing of target display: the next target is shown while the last one is still visible (overlap condition), has disappeared for a certain duration (gap) or disappeared at the same time (synchronous condition). This test is informative because saccade kinematics are very stereotyped and simple analyses can be done to characterize saccadic eye movements. In the next sections, we will first show the main sequence to characterize leftward and rightward average saccadic behavior. After, we will present a typical rightward and a typical leftward saccade done by the patient with particularly large overshoots. Then we will present saccadic movements with horizontal oscillations made by the patient. Finally, from the behavioral observations we made, we then show how small adjustments to the model’s parameters cause it to change from a healthy configuration to a configuration that reproduces the majority of the patient’s conditions.

#### Main sequence

**Figure 4:**
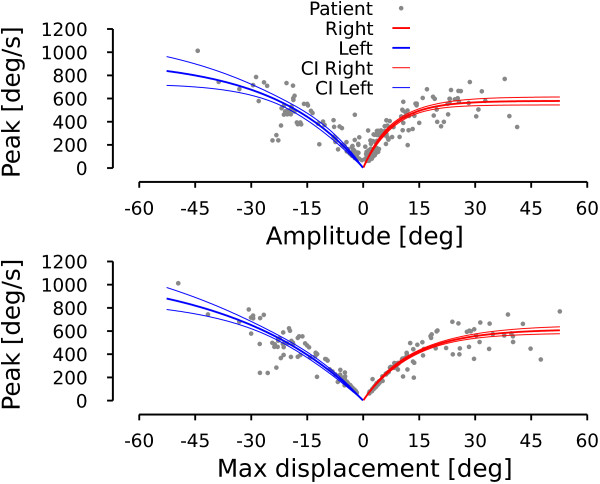
**Patient main sequence.** Upper row represents saccade peak velocity as a function of saccade amplitude. Lower row represents saccade peak velocity as a function of saccadic maximum displacement. Gray dots correspond to the patient data. Thick colored lines represent the average behavior computed with an exponential fit. Thin colored lines represent the 95% confidence interval around the exponential fit. Red lines are used for rightward movements. Blue lines are used for leftward movements. Negative amplitudes (maximum displacements) correspond to leftward movements. Positive amplitudes (maximum displacements) represent rightward movements.

**Figure 5:**
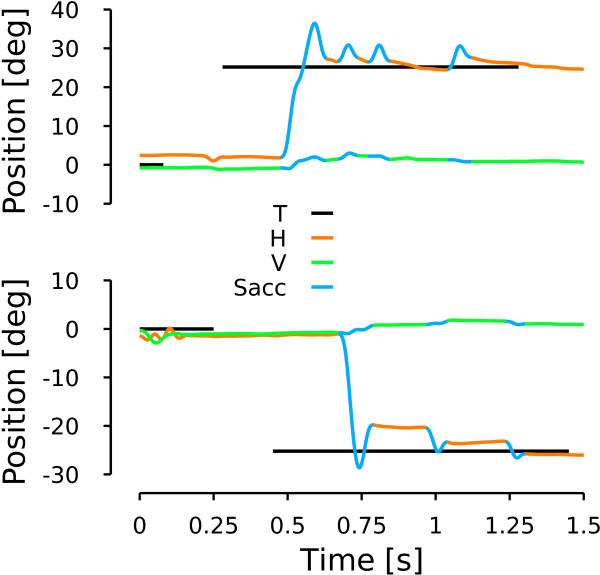
**Patient saccades.** Upper (lower) row represents the time course of eye position when the patient looked at a 25 deg target located on the right (left). Black lines represent target position. Orange lines represent horizontal eye movements. Green lines represent vertical eye position. Blue lines represent saccades. a represents the onset of the first saccade. b represents the hook extrema. c represents the offset of the saccade.

In this section, we characterize the general saccadic behavior of the patient. The upper graph in Figure 4 shows the relationship between saccade amplitude (e.g. difference between eye position at point c and eye position at point b in Figure 5 for the first saccade) and saccade peak velocity known as the main sequence [23]. Leftward (rightward) saccades are represented with a negative (positive) amplitude. Lower graph shows the peak velocity as a function of the maximum displacement during the saccade. The maximum displacement is defined as the difference between eye position at the extrema of the position hook (reversal of the saccadic trajectory, e.g. point b in Figure 5 for the first saccade) and the eye position at the onset of the saccade (e.g. point a in Figure 5 for the first saccade). One can see that the dispersion is smaller for the peak-velocity vs. maximum-displacement relationship than for the peak-velocity vs. saccade-amplitude relationship. To test this, we fitted an exponential model to characterize the different relationships in Figure 4: 

(4) $$\begin{array}{rl} V_{R,\text{Max}} & =(579\pm 35)*\left(1-e^{-(0.128\pm 0.018)A_{R}}\right)\\ \text{MSE} & =5643, \end{array}$$

(5) $$\begin{array}{rl} V_{L,\text{Max}} & =(938\pm 237)*\left(1-e^{(0.043\pm 0.017)A_{L}}\right)\\ \text{MSE} & =11562, \end{array}$$

(6) $$\begin{array}{rl} V_{R,\text{Max}} & =(617\pm 35)*\left(1-e^{-(0.077\pm 0.009)M_{R}}\right)\\ \text{MSE} & =3517, \end{array}$$

(7) $$\begin{array}{rl} V_{L,\text{Max}} & =(1133\pm 303)*\left(1-e^{(0.029\pm 0.011)M_{L}}\right)\\ \text{MSE} & =7400. \end{array}$$

The parameters of each fit is given with their 95% confidence interval. *A*_*R*_ (*A*_*L*_) corresponds to the amplitude of rightward (leftward) saccades. *M*_*R*_ (*M*_*L*_) corresponds to the maximum displacement of rightward (leftward) saccades. *V*_*R*,*Max*_ (*V*_*L*,*Max*_) represents the peak velocity during rightward (leftward) saccades. Finally, MSE represents the mean squared error of each fit. Fits (4)-(7) are shown in Figure 4 using blue thick lines for leftward fits and red thick lines for rightward fits. The 95% confidence interval for each fit is represented in Figure 4 by the corresponding thin lines.

The main sequence expresses that saccadic eye velocity saturates for large-amplitude saccades. For a healthy subject, there is no statistically significant difference between the saturation velocity for leftward and rightward saccades. For our patient, there is an asymmetry between the peak velocity for leftward and rightward saccades: rightward saccades have a velocity saturation approximately half the size of that of leftward saccades. Normal subject have a peak eye velocity saturating between 500 and 700 deg/s [24]. Therefore, this asymmetry does not arise from slow rightward saccadic movements but from extremely fast leftward saccadic movements. This is the first characteristic that the model should reproduce.

Comparing MSEs between equations (4) and (6) and between equations (5) and (7), fits using the maximum displacement as the independent parameter explain more variability than those using saccadic amplitude as the independent parameter. Two-tailed f-tests between residual distributions indicate that this difference between MSEs is statistically significant (leftward saccades: F(163,163)=1.606, p <0.05. Rightward saccades: F(103,103)=1.526, p <0.05). The better fit using the maximum amplitude suggests to us that the command sent to the burst neurons has a normal saccadic shape but that the discharge that stops the saccade is too large.

#### Dynamic overshoot

A saccadic dynamic overshoot corresponds to a fast reversal of the saccadic trajectory before the end of the movement. It is different from a pulse-step mismatch because of the time course of the reversal movement (see [35] for a study of pulse-step mismatch). This can be observed in Figure 5. To characterize the dynamic saccadic overshoot of the patient, we computed a linear regression between rightward and leftward maximum displacements and saccadic amplitudes:

(8) $$\begin{array}{rl} A_{R} & =(0.790\pm 0.013)M_{R}-(0.698\pm 0.2129)\\ R^{2} & =0.96,\;\;p<0.001, \end{array}$$

(9) $$\begin{array}{ll} A_{L} & =(0.928\pm 0.018)M_{L}-(0.528\pm 0.086)\\ R^{2} & =0.98,\;\;p<0.001. \end{array}$$

Regressions (8) and (9) show that the dynamic overshoot made by the patient corresponded to ≈21% of the maximum displacement for rightward saccades and to ≈7% of the maximum displacement for leftward saccades. The coefficient of variation of the two regressions shows that a linear relationship accurately captures the relationship between the maximum displacement and the amplitude of the saccade. The dynamic overshoot is the second major characteristic that the model should simulate.

#### Post-saccadic drift

Figure 5 shows 25 deg rightward (upper row) and leftward (bottom row) saccades made by the patient during the second session. Target position is represented in black, horizontal eye position in orange and vertical eye position in green. Saccades as determined by our algorithm are colored in blue. As shown in Figure 5, a position hook was visible at the end of each saccadic trajectory. The overshoot of the first rightward saccade in Figure 5 has an amplitude of 9.05 deg. The overshoot of the first leftward saccade in Figure 5 has an amplitude of 8.86 deg. Additionally, when saccades were directed rightward, the patient drifted towards the center but this drift was strongly reduced (even absent sometimes) when the patient executed leftward saccades. To quantify the time constant and the amplitude of the drift, we fitted an exponential function to the movements between two saccades. We excluded fits with a time constant larger than 20 seconds and consider them as non-decaying (no rightward movements, 5 leftward movements). The average time constant for the drifts following rightward saccades was equal to 90 ±75 ms and for the drifts following leftward saccades was equal to 142 ±171 ms. The average amplitude of the drifts following rightward saccades was equal to 3.4 ±1.9 deg (statistically different from zero, two-tailed t-test, t(63)=14.22, p <0.001). The average amplitude of the drifts following leftward saccades was equal to -0.5 ±0.9 deg (statistically different from zero, two-tailed t-test, t(60)=-4.60, p <0.001). Finally, we computed the correlation between the amplitude of the drifts and the orbital position at the onset of the drift. We found a significant positive correlation between the amplitude of the rightward drifts and the orbital position (*ρ*=0.418, p <0.001). In contrast, no significant correlation was observed between the amplitude of leftward drifts and the orbital position (*ρ*=-0.098, p=0.450). These statistical analyses confirmed that there was a strong drift following rightward saccades and a marginal leftward drift following leftward saccades. The asymmetrical drift is the third major characteristic of the patient’s saccadic behavior that the model should simulate.

#### Saccadic oscillation

**Figure 6:**
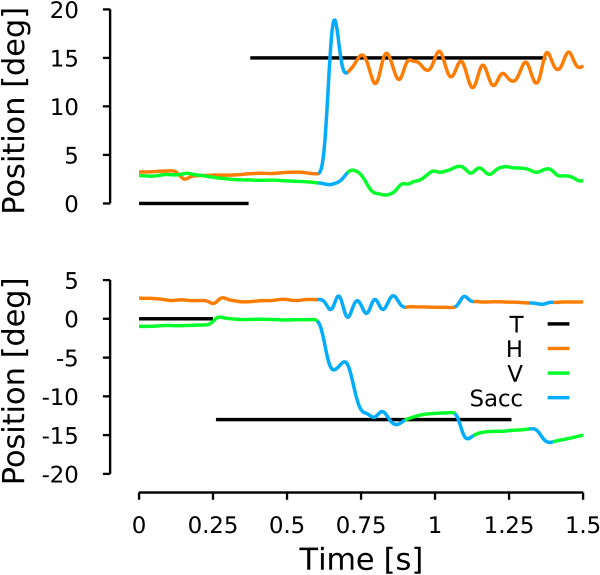
**Patient saccadic oscillation.** Upper row shows a horizontal saccade towards a target located 15 deg to the right. Lower row represents a vertical downward saccade towards a target located 13 deg below the central fixation point. Same color conventions as in Figure 5.

Figure 6, upper panel, shows a horizontal saccade towards a target located 15 deg on the right. As for the rightward saccade in Figure 5, this saccade has a hook in the position trace at the end of the movement. However, unlike the case of Figure 5, the eyes started to oscillate horizontally at the end of the saccade. No oscillations were observed on the vertical channel. To test if the oscillation was linked to the saccadic command, we tested the behavior during saccades. The bottom row of Figure 6 shows a vertical saccade towards a downward 13 deg target. No oscillations were observed on the vertical channel at the end of the saccade. However, horizontal oscillations were observed during the largest vertical saccade. These two behavioral observations indicate an oscillation mechanism based on the cross-inhibition of the short lead inhibitory burst neurons similar to the one previously reported by [6]. We quantified the frequency of the horizontal oscillations following horizontal saccades and during vertical saccades. We computed the oscillation frequency based on the time between successive peak positions during the oscillations. The average frequency in our patient is equal to 14.5 ±3.4 Hz after horizontal saccades and to 13.1 ±3.1 Hz during vertical saccades. We found no significant difference between the two frequency ranges (two-tailed t-test, t(68)=-1.459, p=0.142), pointing towards an identical mechanism in both cases. As in [6], those oscillations are only possible if the omnipause neurons are held off. Therefore, the oscillatory saccadic behavior of the patient indicates that OPN are not reactivated correctly at saccade offset. This behavior is the fourth major characteristic that model should reproduce.

### Model simulations

In this section, we will explain how we reproduced the four major characteristics of the patient saccadic behavior: the dynamic overshoot, the pronounced rightward drift and the attenuated leftward one, the saccadic oscillations and the asymmetry in the peak velocity. First we present how the model can reproduce general characteristics of healthy saccades.

#### Healthy saccade

To simulate healthy human subjects, we tuned the parameters of the model (*cSC*_*Max*_, *cFN*_*Max*_, *iFN*_*o*_ and *iFN*_*Max*_, see methods) to reproduce the main sequence represented by equation:

(10) $$\begin{array}{ll} V_{\text{Max}}\;\;=\;\;\text{sign}(A)\cdot 601.4\cdot \left(1-e^{-0.103∥A∥}\right) & \; \end{array}$$

This main sequence is extracted from a fit we performed on the data presented in Figure 1 of [24]. Compared to the patient situation, there is only one expression of the main sequence because there is neither a dynamic overshoot nor a left-right asymmetry. Thus *A* in eq. (10) corresponds to the amplitude of the saccade, whether it is rightward or leftward. To account for the natural undershoot behavior of saccades, we set a gain of 0.9 for the saccadic displacement.

**Figure 7:**
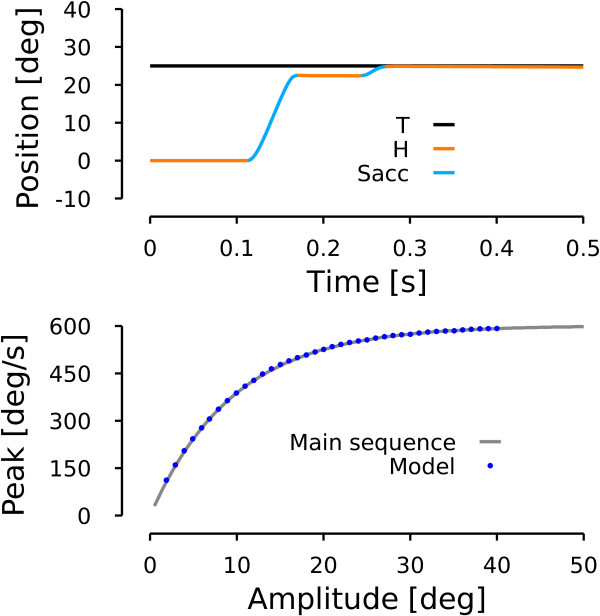
**Model simulation: healthy subject.** Upper row represents a simulation (time course of horizontal eye position) of the model when a target is presented 25 deg to the right. Same color conventions as in Figures 5–6. Lower row represents simulation of a main sequence by the model. The gray line represents a fit computed on data extracted from Figure 1 of [24]. The blue dots represent simulations of the model for a range of amplitudes between two and 40 deg in steps of one deg.

The upper panel of Figure 7 shows the time course of a simulated saccadic movement toward a rightward 25 deg target. Because of the undershoot, the model generated two saccades. The first saccade ended at 22.5 deg (peak velocity: 545 deg/s) and a corrective saccade of 2.5 deg (peak velocity: 138.5 deg/s) was triggered by the model of the cortex to cancel the remaining visual error.

The solid gray line in the lower panel of Figure 7 represents the main sequence of eq. (10) while the blue dots represent simulated saccades with a range of amplitudes between two and 40 degrees in steps of one degree. This panel shows that, once tuned, the model reproduced correctly the desired behavior.

#### Patient simulation of average behavior: asymmetric peak velocity and main sequence

To reproduce the main sequence of the patient, we increased the activity of the contralateral caudal fastigial nucleus and the contralateral caudal superior colliculus (*cFN*_*Max*, *patient*_>*cFN*_*Max*, *healthy*_ and *cSC*_*Max*, *patient*_>*cSC*_*Max*, *healthy*_). Those parameters were tuned to reproduce the peak velocity-maximum displacement relationship presented in Figure 6. To simulate the drift, first we increased bilaterally the time constant of the NPH (from 20 seconds to 22.5 seconds). Second, we increased the gain of the projection from the right EBN and left IBN to the right abducens nucleus (step gain from 0.15 to 0.185). This second modification disturbs the compensation of the longest time constant of the eye plant on one side, and thus generates an asymmetrical drifting behavior as observed in the patient. It must be stressed that the effect of the drift could not be observed in the main sequence but was present in the patient behavior. Thus, we already included the drift modifications in those simulations but the results of the changes will be discussed in the next section. To reproduce the dynamic overshoot, we increased the maximum discharge of the ipsilateral caudal fastigial nucleus (*iFN*_*Max*, *patient*_>*iFN*_*Max*, *healthy*_) and we triggered the ipsilateral caudal fastigial nucleus activity sooner (*iFN*_*o*, *patient*_<*iFN*_*o*, *healthy*_). Through those changes, the ipsilateral EBNs start to discharge too soon, and thus reverse the movement. The higher and/or sooner the ipsilateral caudal fastigial activity, the bigger the saccadic overshoot made by the model. Each of the piecewise functions was tuned independently for leftward and rightward movements to match the dynamic overshoot amplitude for leftward and rightward saccades expressed by eq. (8) and (9).

**Figure 8:**
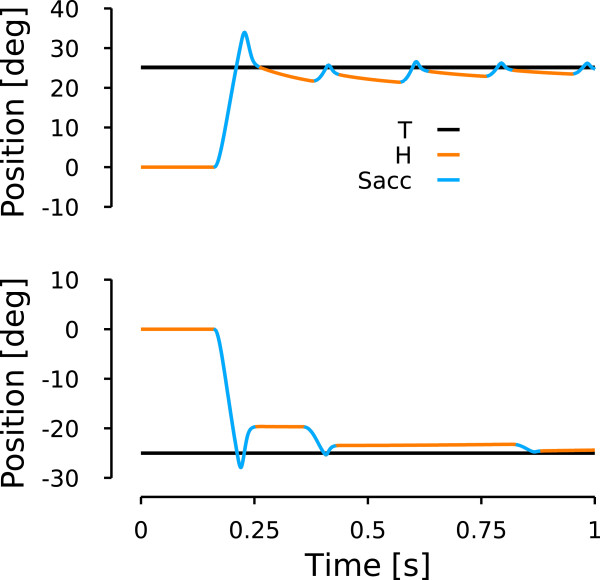
**Model simulation: patient extreme conditions.** Upper row represents how the model reproduces specific movements show in Figure 5. Same layout and color conventions as in Figure 5.

**Figure 9:**
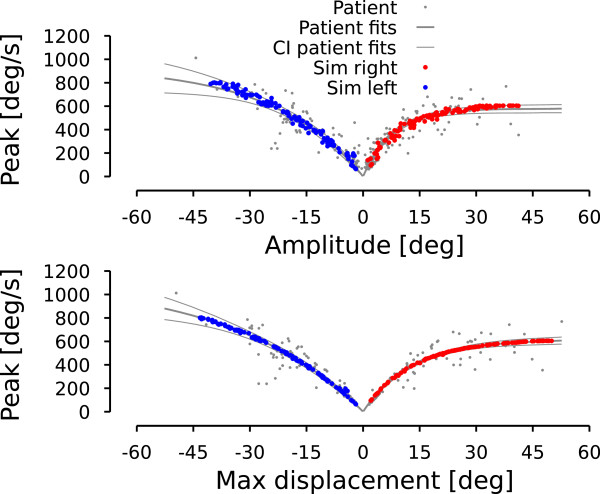
**Model simulation: patient saccadic average behavior.** Superimposed on the patient data of Figure 4, this figure shows how the model reproduces the average behavior of the patient. Red dots represent rightward simulated saccades. Blue dots represent leftward simulated saccades. Same layout as in Figure 4.

Once the parameters were tuned to match this relationship, we added a 25% random gaussian noise on *iFN*_*Max*, *patient*_ to account for a part of the variability observed in the patient data (noise amplitude arbitrarily chosen). Then, we simulated 117 leftward and 117 rightward saccades with varying amplitudes between 2 and 45 deg. Figure 8 shows the main sequences generated by the model. Upper panel of Figure 9 represents the saccade-amplitude vs. peak-velocity relationship while the lower panel shows the peak-velocity vs. maximum-displacement relationship. Red dots represent the rightward saccade simulations and blue dots represent the leftward saccade simulations. As expected by the tuning of the parameters, the model reproduces correctly the maximum displacement-peak velocity relationship.

Finally, we computed the regression between the maximum displacement and the saccade amplitude for leftward and rightward simulated saccades:

(11) $$\begin{array}{rl} A_{R,S} & =(0.773\pm 0.014)M_{R,S}-(0.611\pm 0.436)\\ R^{2} & =0.98,\;\;p<0.001, \end{array}$$

(12) $$\begin{array}{rl} A_{L,S} & =(0.936\pm 0.005)M_{L,S}-(1.098\pm 0.138)\\ R^{2} & =0.99,\;\;p<0.001. \end{array}$$

Comparing regressions (8)-(9) with regressions (11)-(12), one can see that the model correctly approximates the patient behavior. A t-test showed that the slope of eq. (8) is not statistically different from the slope of eq. (11) for rightward saccades (two tailed t-test. t(115)=0.4513, p=0.3264). Similarly for leftward saccades, a t-test showed that the slope of eq. (9) is not statistically different from the slope of eq. (12) for rightward saccades (two tailed t-test, t(115)=0.8768, p=0.1909).

#### Patient simulation: asymmetric drift and dynamic overshoot

Simulating the average behavior of the patient is important for the model, but it is also important to show that it can reproduce extreme conditions. The examples of Figure 5 present a fairly large dynamic overshoot for the first saccade compared to the average behavior. Therefore, we tuned the model with a new set of the four parameters (*cSC*_*Max*_, *cFN*_*Max*_, *iFN*_*o*_ and *iFN*_*Max*_) to reproduce the larger dynamic overshoot of the first saccades of the trials in Figure 5. To reproduce the patient behavior, we used the behavioral observation that the main sequence is better defined if one used the maximum displacement instead of the saccadic displacement. Therefore, the parameters were tuned as a function of the maximum displacement instead of the saccadic amplitude. We used the inverse of relationships (8) and (9) to compute the amplitude of the saccade sent to the cerebellum and the colliculus by the cortex. All the other parameters were kept constant. Figure 8 shows a rightward simulated saccade (upper panel) and a leftward simulated saccade (bottom panel) that reproduce the patient behavior presented in Figure 5: the dynamic overshoot in both directions and the asymmetric drift at the end of the movement. The upper panel shows the simulation of a rightward saccade toward a 25 deg visual target. The saccadic gain in the cortex was set to 1 to reproduce the behavior shown in Figure 5. Compared to the upper panel of Figure 5, one can see that the general behavior is reproduced: the first saccade overshoots the target and subsequent saccades are triggered even though the eye is close to the target. In addition, a drift can be observed between rightward saccades. The lower panel shows the simulation of a saccade towards a visual target located 25 deg on the left of the center. For this leftward movement, the saccadic gain in the cortex was set to 0.8. Comparing this simulation with the patient behavior presented in the bottom panel of Figure 5, one can see that the model reproduces correctly the desired behavior. The drift between the saccades is greatly reduced compared to rightward movements but the dynamic overshoot is still present. The amplitude of the dynamic overshoot of the first saccade is identical in the simulations (rightward simulation: 9.1 deg, leftward simulation: 8.3 deg) compared to the ones observed in Figure 5.

#### Patient simulation: saccadic oscillations

**Figure 10:**
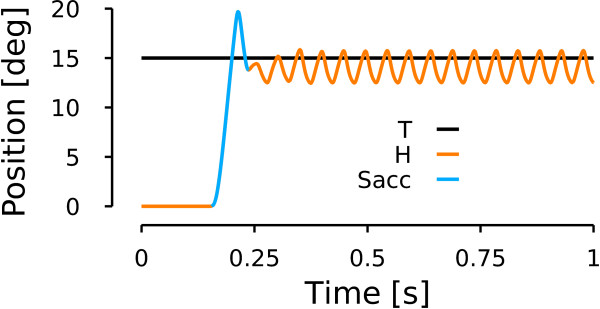
**Model simulation: saccadic oscillations.** Simulation of a rightward horizontal towards a target located 15 deg on the right of the central fixation point. Same color conventions as in Figure 6.

Figure 10 shows the model behavior when the OPN activity is not reactivated at the end of a 15 deg rightward saccade (to reproduce the patient behavior in the upper panel of Figure 6). The model starts to oscillate if the OPN are not reactivated at saccade offset. The simulated oscillation mechanism is similar to the one reported in [6] and can be reproduced by the model because of the cross-inhibition of the short-lead inhibitory burst neurons and the post-inhibitory rebound of the neurons. To generate the oscillation pattern of Figure 10, we decreased only the input gain of the OPN and we kept all the other average parameters as in Figure 9. Therefore, at the end of the saccade when the OPN should have fired to prevent the sIBN_L_- sIBN_R_ circuit from oscillating, the OPN inhibition by the long-lead inhibitory burst neurons could not be stopped and an oscillatory movement started. The main differences between our simulation and the patient observation is the variable amplitude of the oscillations. To generate a variable amplitude of the oscillations, we could included some variability in the amplitude of the input gain of the OPN, but that is beyond the scope of this paper. The patient also exhibited shorter oscillatory periods. To simulate those situations, the input OPN gain must be amplified sooner. This will excite the OPN and stop the oscillations.

## Discussion

Our patient had several ocular motor abnormalities: saccades were asymmetric and had increased peak velocity, a dynamic overshoot, an asymmetrical postsaccadic centripetal drift and saccade-induced oscillations.

To account for these observations, we focused on a neuromimetic model of saccadic control with a detailed representation of the brainstem. A key point while designing a model is that several theoretical control structures could be built to reproduce our patient’s behavior. However, the pathophysiological consequences linked to a modification of the model make sense only if the model structure relates closely to the actual neural circuitry. With that point in mind, we built a model that is constrained by the current knowledge of the anatomy (connections between neural structures) and the neurophysiology (average discharge of the neural structures). Once the model architecture was defined, we tuned the model parameters to reproduce the saccadic behavior of healthy human subjects. Then we modified just 8 out of the 112 model parameters to reproduce our patient’s saccadic eye movements (each one of the modified parameters being a piecewise linear function of saccade amplitude).

If the model it to help us understand the complex interactions between the different subparts of the system, the likelihood of the simulated deficit must always be put in perspective with other studies of the neuronal structure assumed to be defective. The interpretation of the changes applied to the model to simulate our patient’s eye movements is important because abnormal behavior could have different functional or anatomical origins. In addition, the patient’s behavior could arise from a focal lesion or be the resultant of several defects. Once a set of parameters has been found that mimics the deficit in the behavior, one must ask how likely the changes in the parameters are to reflect the actual neuronal defects in the patient.

The first deficit we simulated with our model was the asymmetrical peak velocity vs. saccade amplitude relationship of our patient. Saccade kinematics in the model can be tuned using four parameters: the maximum collicular discharge, the maximum contralateral cerebellar discharge, the maximum ipsilateral cerebellar discharge and the onset timing of the ipsilateral cerebellar discharge. The saccadic peak velocity can be increased in the model by increasing the maximum collicular discharge and the maximum contralateral cerebellar discharge. Because the collicular discharge is shaped by the cerebellar activity through the disfacilitation signal, an increase of the maximum collicular discharge is equivalent to a decrease of the disfacilitation gain from the cerebellum. Therefore, the patient’s abnormal saccadic peak velocity can also be explained by a cerebellar defect. However, the colliculus is not inhibited only by the cerebellum; another major source of inhibition comes from the substantia nigra pars reticulata (SNr) [36]. Therefore, the decrease of collicular inhibition (and thus the increase of collicular discharge leading to an increased saccadic peak velocity) could also be explained by a deficient SNr. In addition, clinical examinations of the patient showed a deficit in the nigrostriatal pathways. However, Vidailhet et al. have reported that patients with striatonigral deficits have close to normal saccades [37]. Thus, the abnormal eye movements of our patient are unlikely to arise from a low inhibition on the colliculus from the SNr and we favor a cerebellar origin.

The second deficit we tried to reproduce with the model was the dynamic overshoot of the patient. Optican and colleagues have proposed that the ipsilateral cerebellar discharge is responsible for stopping the saccade through a choke signal sent to the contralateral EBN and IBN [13,14]. Hence, to model the dynamic overshoot of our patient, we increased the maximum discharge of the ipsilateral cerebellar discharge and triggered its activity sooner. These changes increased the choke drive sent to the contralateral EBN and IBN and reversed the direction of the saccade. Thus, the dynamic overshoot of our patient can also be explained by cerebellar dysfunction.

The third deficit of our patient that our model sought to simulate was her asymmetrical post-saccadic drifting behavior. We changed unilaterally the gain of EBN and IBN projections at the input of the right abducens nucleus and by slightly increasing bilaterally the NPH time constant to reproduce the drift. The NPH changes reflect a pathological neural integrator that could be explained by a loss of compensation by the cerebellum [34,38,39].

Thus, the first three deficits in this patient could be accounted for by changing a minimal subset of model parameters, *all of which could be linked to a cerebellar problem*.

Finally, we showed how a simple decrease of the input gain of the OPN allowed us to reproduce the oscillatory behavior of the patient (her fourth deficit). (As pointed out in the result, we chose a sustained oscillation as an example because they are harder to generate.) If one wants to stop the oscillations sooner, it can easily be done by restoring the input gain to its normal state. The change of the input gain can reflect either a deficit of the OPN or a deficit of the excitatory drive sent to the OPN to reactivate them. It is known that the OPN receive projections from the superior colliculus [40]-[42] and from the cerebellum [13,14]. Consequently, if one or both of those two structures does not send an appropriate signal to the OPN, they will not be reactivated and an oscillation will be triggered. Therefore, the oscillations may be attributable to a cerebellar dysfunction.

In the present study, the first step was to acquire accurate recordings for analysis and identification of the patient’s ocular motor impairments, especially when those could not be easily identified by a clinical examination (such as increased leftward saccade velocities, dynamic overshoot and saccadic oscillations). In the next steps, the model simulation showed that the entire ocular motor behavior could be reproduced by the alteration of intra-cerebellar structures. This result is consistent with the marked cerebellar syndrome exhibited by our patient. A second interesting outcome of this simulation was that this diverse ocular motor behavior could be induced by subtle adjustments of a limited number of parameters.

Our model reproduces the key abnormal saccadic ocular motor deficits of our patient, even though it was built with some limitations in mind. We did not include simulations of saccades smaller than one degree because we did not have enough data to analyze the behavior of our patient for such small amplitudes. A second simplification comes from the constant saccadic gain over the whole range of amplitudes. Adding a dependency of the saccadic gain as a function of amplitude would not help us link a neural deficit to the patient’s behavior. Therefore, we decided to simplify the amplitude vs. gain relationship as a constant. In addition, the model of the cortex is very simple. However, the brainstem connectivity reproduces as closely as possible (for a lumped model) the known anatomical connectivity and the functions of the ocular motor brainstem. This level of detail in the model was sufficient to reproduce the patient’s behavior. Therefore, we think that it could be used also to reproduce other saccade-related dysfunctions (congenital nystagmus, saccadic intrusions, etc) that do not depend on defects in the cerebrum. Thus, our cortical model is adequate to serve its sole purpose: sending desired saccade displacements to the subcortical areas involved in the control of saccade trajectory. Other models have been proposed that include more functionally detailed cortical areas that trigger saccades and pursuit movements [43]-[50]. Those models provided assumptions on the mechanisms from which arise gaze-evoked nystagmus [43], myasthenic disease [44], latent/manifest latent nystagmus [45] and congenital nystagmus [46]-[50]. They also provided predictions on the patient’s behavior on the interactions between saccades, pursuit and fixation. Although they reproduce a lot of complex functions, they do not incorporate an accurate description of the bilateral ocular motor brainstem connectivity and a model of neuron behavior. Therefore, a major difference arises between our new model (and the models of [4,6,8]) and more functionally-built models [43]-[50] in that the properties of the saccadic movements emerge from the connectivity, not from the design of the functions included in the model. We think that these approaches are complimentary.

In conclusion, a lumped neuromimetic model of brainstem eye movement circuitry enabled us to propose that all of this patient’s diverse deficits could be localized to a single structure, the cerebellum. Future work should be aimed at extending such models to include other functions in other parts of the brain, while maintaining the simplicity of interpretation given by the lumped circuitry. Such models would have further specificity and increase their utility in clinical diagnosis.

## Endnote

^a^For the sake of simplicity, “contralateral (ipsilateral)” will be used instead of “contralateral (ipsilateral) to the saccade” in the rest of the text.

## Competing interests

The authors declare that they have no competing interests.

## Authors’ contributions

PMD and LMO made the analyses, built the model, made the graphs, wrote the methods and results sections. BG and ER diagnosed the patient, recorded her eye movements, wrote the discussion and reviewed the document. PP wrote the introduction and reviewed the document. All authors read and approved the final manuscript.
